# Factors associated with the survival of adults with COVID-19 using a high-flow nasal cannula in a tertiary hospital in northern Peru during the second wave of the pandemic

**DOI:** 10.1371/journal.pone.0309855

**Published:** 2025-04-16

**Authors:** Edwin Aguirre-Milachay, Darwin A. León-Figueroa, Cristian Díaz-Vélez, Mario J. Valladares-Garrido

**Affiliations:** 1 Hospital Nacional Almanzor Aguinaga Asenjo, Chiclayo, Peru; 2 Facultad de Medicina Humana, Universidad de San Martín de Porres, Chiclayo, Peru; 3 Hospital Nacional Sergio E. Bernales, Lima, Perú; 4 Facultad de Medicina Humana, Universidad Andina del Cusco, Cusco, Perú; 5 Escuela de Medicina Humana, Universidad Señor de Sipán, Chiclayo, Peru; Kaohsuing Medical University Hospital, TAIWAN

## Abstract

**Objectives:**

To identify factors associated with survival in patients admitted to the intensive care unit (ICU) for COVID-19 who used high-flow nasal cannula (HFNC) in a tertiary hospital in northern Peru during March to May 2021.

**Methodology:**

A retrospective observational cohort study was carried out, including medical records selected according to established inclusion criteria. The dependent variable was survival, measured in days from admission to hospital discharge or death. Factors associated with survival related to demographic, clinical, laboratory, and imaging characteristics were investigated, as well as treatment-related parameters and variables associated with the use of HFNC. Hazard ratios (HR) were estimated to identify independent risk factors associated with survival.

**Results:**

Of 154 patients, the mean age was 58.29 years. The most frequent comorbidities were arterial hypertension (29.2%), diabetes mellitus (20.6%), and obesity (17.4%). The median time of HFNC use was 5 days (interquartile range: 3–9 days). It was found that 32.2% of the patients required mechanical ventilation, and 51.6% died. The mean time of mechanical ventilation use was 15.1 ± 13.3 days. Survival was 97.5% at 48 hours, 85% at 7 days, 62% at 14 days, and 16.3% at the end of the study. Variables decreasing survival in patients with COVID-19 who were users of NFVC were age ≥ 60 years (HR = 2.23; 95% CI: 1.21–4.08), presence of arterial hypertension (HR = 1.87; 95% CI: 1.01–3.45), increased work of breathing on hospital admission (HR = 2.38; 95% CI: 1.31–4.35), and a ROX index (iROX) < 3.85 (HR = 1.71; 95% CI: 1.01–2.93).

**Conclusions:**

Factors associated with survival were arterial hypertension and iROX < 3.85 with a mortality hazard of 1.5 times, age older than 60 years, and respiratory effort scale at admission WOB ≥ 4 points with more than twice the mortality hazard. The results of this study highlight the importance of early and accurate assessment of risk factors in patients with COVID-19 who use HFNC. Identifying these factors can help clinicians make more informed decisions and prioritize interventions that could potentially improve survival in this group of patients.

## Introduction

The COVID-19 pandemic, declared by the World Health Organization (WHO) on March 11, 2020, and valid until May 5, 2023, created a public health emergency of international importance. This period challenged health systems worldwide, highlighted by a significant increase in the severity and mortality of cases [1,2]. Globally, as of July 7, 2024, more than 775 million cases have been confirmed, with clinical manifestations ranging from mild to severe, and more than 7 million deaths have been recorded [3].

At the onset of the COVID-19 pandemic, clinical practice guidelines for the new coronavirus were not yet available, and all public and private health systems in Peru were overwhelmed with cases of severe atypical pneumonia, resulting in high oxygen demand, hospitalizations, and intensive care unit (ICU) admissions [4–6]. In a hospital in Lima, the capital of Peru, the case fatality rate at ICU admission was 62%, exceeding the world average of 37% reported in a meta-analysis in 2020 [7,8]. Severe symptoms of COVID-19 occurred in 20% of infected persons, and one-fifth of these developed acute respiratory distress syndrome (ARDS) or acute respiratory failure (ARF), requiring ICU care and oxygen support [9].

In 2020, there were still doubts about oxygen management in patients with ARDS due to COVID-19. One study showed that adequate ventilatory support improved survival time [10]. Worldwide, 88% of ICU patients received invasive mechanical ventilation (IMV), while only 11% received non-invasive mechanical ventilation (NIV) [11]. A meta-analysis from the same year showed that NIV reduced mortality compared to conventional oxygen therapy [12].

In this context, the use of high-flow nasal cannula (HFNC) has emerged as an important alternative in the management of patients with ARF [13]. This device offers advantages such as decreased dead space with the consequent elimination of CO2, generation of positive airway pressure, increased circulating volume, reduced work of breathing, and improved mucociliary transport [14–16]. This improves oxygenation and reduces the need for IMV [13].

The use of HFNC currently plays an important role in the management of ARDS in both COVID and non-COVID pathology, and systematic reviews indicate that its efficacy is comparable to that of non-invasive ventilation (NIV). Additionally, it is associated with fewer adverse effects compared to NIV, which improves patient comfort and outcomes [17,18]. Although it may not be suitable for those patients with severe hypoxemia or other complicating factors. Despite that, there are no Peruvian studies in adults that evaluate data on the management of this device during the pandemic and post-pandemic.

The aim of our study is to identify factors associated with the survival of patients admitted to the ICU for COVID-19 who used HFNC in a tertiary hospital in northern Peru during the second wave. Understanding these factors is essential to maximize the efficacy of HFNC and improve clinical outcomes in resource-limited settings, as well as providing us with a perspective on the population that was managed during the pandemic in the pre-vaccination period. Through a thorough analysis, this study provides valuable information that will facilitate the creation of more effective strategies for the management of COVID-19 and likely non-COVID-19 pathologies in similar hospital settings, and it may even add value to specific oxygenation and ventilation scales in these pathologies [13].

## Materials and methods

### Study design

A retrospective cohort study was conducted in patients diagnosed with SARS-CoV-2 infection admitted to the Hospital Nacional Almanzor Aguinaga Asenjo (HNAAA), located in the Lambayeque region, Peru, during the period from March to May 2021.

### Eligibility criteria

Patients with a diagnosis of COVID-19 confirmed by a serological rapid test (IgM and/or IgG), a molecular test by reverse transcriptase polymerase chain reaction (RT-PCR), or an antigenic test who were hospitalized at HNAAA between March and May 2021 were included in the study. Incomplete medical records that did not include the recording of outcome variables were excluded.

### Population, sample, and sampling

The study population consisted of 155 patients admitted to a general ward and/or ICU at HNAAA with a confirmed diagnosis of COVID-19 who used HFNC during the study period, between March and May 2021. The second pandemic wave of COVID-19 in Peru occurred during this period.

The sample was obtained using the EPIDAT v4.2 program database, considering a survival study design with the following parameters: significance level of 95%, power of 90%, percentage exposed of 59.2% (variable defined by HACOR greater than 5 at 48 hours of NIV), and a HR of 4.33. A sample of 40 patients (20 exposed and 20 unexposed) was determined [19]. We worked with a sample population because it was larger than the established sample size.

A non-probabilistic census-type sampling was performed among patients admitted to a general ward and ICU for COVID-19 who used HFNC in the HNAAA during the second wave.

### Variables

Dependent variable: time to outcome, operationally defined as time from hospital admission to death or discharge measured in days.

Independent variables: age, sex, time of illness, waiting time to use HFNC (days), comorbidities, oxygen saturation with oxygen therapy on hospital admission, SatO2/FiO2 index on hospital admission, respiratory frequency on hospital admission, Work of breathing (WOB) respiratory effort scale on hospital admission, percentage of serum lymphocytes, C-reactive protein, lactate dehydrogenase, D-dimer, serum glucose, type of infiltrate, pulmonary involvement, SatO2/FiO2 index within 12–24 hours of HFNC use, WOB work of breathing scale based on respiratory frequency, nasal flaring, use in inspiration of sternocleidomastoid muscle, use of abdominal muscles in expiration measured within 12–24 hours of HFNC use, respiratory rate per minute recorded within 12–24 hours of HFNC use, PaO2/FiO2 ratio measured by blood gas measured within 12–24 hours of HFNC use, IMV criteria defined as the presence of at least one criterion after the use of HFNC (hemodynamic instability, shock, vasopressor requirements, PaO2/FiO2 less than 100, PaC02 greater than 40, increased work of breathing with paradoxical breathing, persistent respiratory rate greater than or equal to 30 per minute), time of IMV use.

### Procedures and techniques

A registry and observation of patients admitted to a general ward of COVID-19 at the HNAAA from March to May 2021 who were users of HFNC were initially carried out, and a database was created with data on the outcome of hospitalization, time in days to outcome, and treatment parameters. Patients requiring mechanical ventilation were transferred to the ICU, while the rest were managed in the general ward under the supervision of critical care. In patients who were admitted to mechanical ventilation, follow-up continued until the outcome.

A data collection form from the electronic medical records was used, which includes the following sections: demographic, clinical, laboratory, and imaging characteristics; treatment-related parameters; and variables related to the use of HFNC.

The research was conducted using a protocol approved by an institutional ethics committee. Permission for data collection was sought from the relevant departments and heads of service. Data collection started on August 22, 2023, and continued until February 10, 2024, using the data collection form with the previously developed database.

### Data analysis plan

The analysis was performed using Stata version 17 software, including univariate, bivariate, and multivariate analyses.

Univariate analysis: Measures of central tendency and dispersion were calculated for quantitative variables that met normality criteria, as well as frequencies and percentages for qualitative variables, based on demographic, clinical, laboratory, imaging, and treatment characteristics.

The Kaplan-Meier method was used to calculate mortality tables, estimating the probability of survival of patients admitted to the ICU for COVID-19 who received NIV with HFNC at HNAAA during the months of March to May 2021.

Bivariate analysis: Parametric data were analyzed using the Student’s t-test for independent samples for quantitative and quanti-qualitative variables, the Chi-square test for nominal qualitative variables, and the Mann-Whitney U test for ordinal variables, considering a significance level of p < 0.05 (95% confidence level). The log-rank test was used for bivariate analysis of time, with a significance level of p < 0.05.

Multivariate analysis: Survival analysis was performed using a Cox proportional hazards regression model to estimate hazard ratios (HR) and their 95% confidence intervals (95%CI) to analyze the prognostic factors associated with survival, taking into account the time to the event of interest. Time to the event of interest (survival) was defined as time from hospital admission to hospital discharge. In the multiple models, the confounding variables that were associated with the simple model were entered; moreover, these variables had to meet the assumptions of independence, survival censoring, and proportionality of hazards through graphical or statistical comparison to be included in the Cox model. Additionally, the Kaplan-Meier survival curve was constructed using the log-rank test. Multicollinearity was evaluated in all the independent variables that entered the final Cox model.

### Ethical considerations

The study protocol was approved by the Ethics Committee of the Hospital Almanzor Aguinaga Asenjo, with code No059-CIEI-RPLAMB-2023. The principles established in the Declaration of Helsinki were complied with. It was not necessary to obtain informed consent from the patients since information from the medical records was used. The collected data were recorded and stored in a database in Excel format, using codes and acronyms to protect patient identity. Only the researchers had access to this information.

## Results

### Epidemiological characteristics of the population

The database consisted of 156 patients; one patient was excluded for not having the mortality outcome, and one patient was censored for not having the time to outcome. A total of 155 patients who used HFNC were studied, and 154 were considered for the final analysis.

Among the epidemiological characteristics, the minimum age was 26, and the maximum was 93 years. The most frequent comorbidities were hypertension, obesity, and diabetes mellitus. We found 2 patients with cardiovascular disease, 1 patient with chronic lung disease, 4 patients with cancer (mortality 3/4), 4 patients with liver cirrhosis (mortality 4/4), and 5 patients with end-stage chronic kidney disease (mortality 4/5).

Among the clinical parameters at hospital admission, saturation on admission without oxygen (O2) was 88.5% (82–90) with a minimum of 54% and a maximum of 97%; most patients had dyspnea (85%); the minimum respiratory frequency was 18, and the maximum was 48/min. The minimum admission SatO2/FiO2 was 42, and the maximum was 260; the minimum admission WOB was 1, and the maximum was 6.

The diagnosis was made with a serological test in 51.1% and with an antigenic test in 48.9%. Within the analytical analysis, the percentage of lymphocytes minimum was 1 and maximum was 44%, PCR minimum was 0.3 and maximum was 154 mg/dl, LDH minimum was 266 and maximum was 1128 mg/dl, D-dimer minimum was 0.25 and maximum was 29 ng/dl, and glucose minimum was 11 and maximum was 735 mg/dl (Table 1).

**Table 1:** Characteristics of COVID-19 patients using high-flow nasal cannula according to mortality.

Variables	Total	Mortality	
	N = 155	Yes	No
Age (years)	58.29±12.9^*^	64.28±10.6^*^	51.92 ±12.1^*^
Sex			
Female	57 (36.8)	34 (59.7)	23 (40.4)
Male	98 (63.2)	46 (46.9)	52 (53.1)
Time of illness (days)^**¥**^	7 (6-10)**	7 (6-10)**	7 (6-10)**
Waiting time to HFNC (days)^**¥**^	2 (1-4)**	2 (1-3)**	2 (1-4)**
**Comorbidities:**			
High blood pressure	45 (29.2)	34 (75.6)	11 (24.4)
Diabetes mellitus	32 (20.6)	19 (59.4)	13 (40.6)
Overweight/Obesity	27 (17.4)	13 (48.2)	14 (51.8)
**Physical examination (hospital admission)**			
Oxygen saturation^**¥**^	93 (89-95)**	92 (88-95)**	94 (91-95)**
SatO2/FiO2	101 (96-105)**	98.5 (93-103)**	104 (100-107)**
Respiratory frequency^**¥**^	26 (23.5-30)**	26 (24-30)**	25 (22-30)**
WOB ≥4 points	68 (54.9)	40 (61.6)	28 (47.5)
**Analytics** ^**¥**^			
Lymphocytes (%)	8 (5-14)**	6.7 (5-9.8)**	10.2 (7-17.7)**
PCR (mg/dl)	10.2 (4.9-18.5)**	11.9 (5.8-23.8)**	9.1 (4.3-16.1)**
LDH (UI/l)	617.3±219.9^*^	701.4±246.9^*^	591.6±190.1^*^
D-Dimer (mcg/ml)	1.2 (0.6-2.6)**	1.58 (0.9-3.25)**	0.7 (0.5-2.1)**
Glucose (mg/dl)	98 (83-124)**	99 (85-127)**	94 (80-115)**
**Type of infiltrate** ^**¥**^			
Tarnished glass	22 (40)	8 (36.4)	14 (63.6)
Cobblestone	2 (3,6)	1(50)	1 (50)
Consolidation	9 (16.4)	3 (33.3)	6 (66.7)
Mixed	22 (40)	10 (45.4)	12 (54.6)
**Lung involvement**			
Mild	4 (5.3)	0	4
Moderate	22 (29.3)	9 (40.9)	13 (59.1)
Severe	49 (65.3)	22 (44.9)	27 (55.1)

* Mean and standard deviation; ** Median and interquartile ranges (25–75%). ¥ Missing values are detailed in variables such as time of illness, saturation on admission with conventional oxygen therapy, respiratory frequency (RF), laboratory, and imaging. Work of breathing (WOB).

### Parameters of treatment with HFNC

The median time of use of HFNC was 5 (3–9) days, with a minimum of 1 and a maximum of 50 days, and the mean oxygen flow was 57.8 ± 14.8 L/min. Within the oxygenatory and ventilatory parameters at 12–24 hours after the start of HFNC, we found a median respiratory frequency of 24 (20–30) with a minimum value of 16 and a maximum of 50; in patients who died, the median respiratory frequency was 28 (22–30). The minimum SatO2/FiO2 value was 32, and the maximum was 317. In the ROX index (iROX), the minimum value was 1.06 and the maximum was 23; in WOB, the minimum value was 1 and the maximum was 6; in PaO2/FiO2, the minimum value was 42 and the maximum was 377 (Table 2).

**Table 2:** Parameters and times related to the use of high-flow nasal cannula and mechanical ventilation according to mortality in patients with COVID-19.

Variables	Total	Mortality	
	n (%)	Yes	No
Oxygen values at 12–24 hours using HFNC			
iROX^¥^	4.4 (3.23-5.83)**	3.86 (2.97-4.55)**	5.33 (4.08-6.8)**
WOB≥4^¥^	60 (42.9)	45 (63.3)	15 (21.7)
PaO2/FiO2^¥^	50 (65-121)**	71 (61-91)**	106.8 (76.9-149) **
SatO2/FiO2	103 (93-126)**	97 (90-105.5)**	108 (100-150) **
Mechanical ventilation criteria	106 (68.4)	80 (100)	26 (34.7)
Time of use of mechanical ventilation	11.5 (6-20)	9 (4-15)	15 (10-30)

* Mean and standard deviation, ** Median and interquartile ranges (25–75%), ¥ Missing values are detailed for variables such as the ROX index (iROX), Work of breathing (WOB), and arterial oxygen pressure/inspired oxygen fraction (PaO2/FiO2).

We found that 106 patients had criteria for mechanical ventilation (68.4%), 50 were admitted to mechanical ventilation (32.2%), and 80 patients died, with a prevalence of 51.6%. The mean time on mechanical ventilation was 15.1 ± 13.3 days (Table 2).

### Survival analysis

The incidence of mortality in patients using HFNC was 7.4 deaths per 100 patient days (5.9–9.3). The mean time to death was 13.45 ± 9.9 days, and the mean time to discharge was 21.5 ± 11.9 days. Survival at 48 hours was 97.5%, at 7 days it was 85%, at 14 days it was 62%, and at the end of the study it was 16.3%.

**Fig 1:**
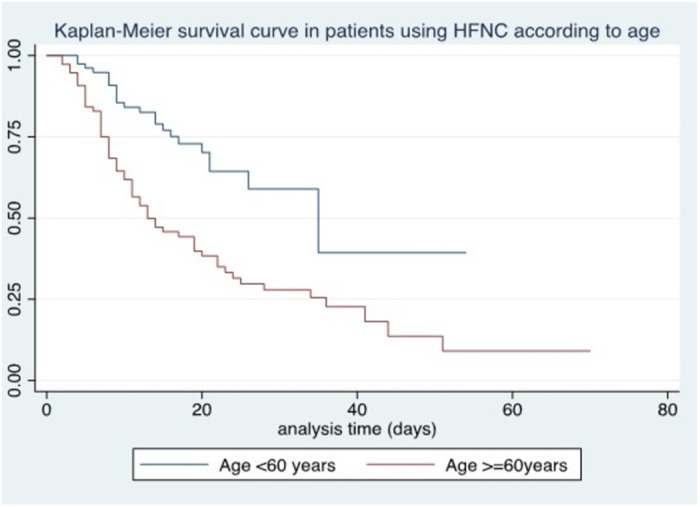
Kaplan-Meier survival curve in patients using HFNC according to age.

**Fig 2:**
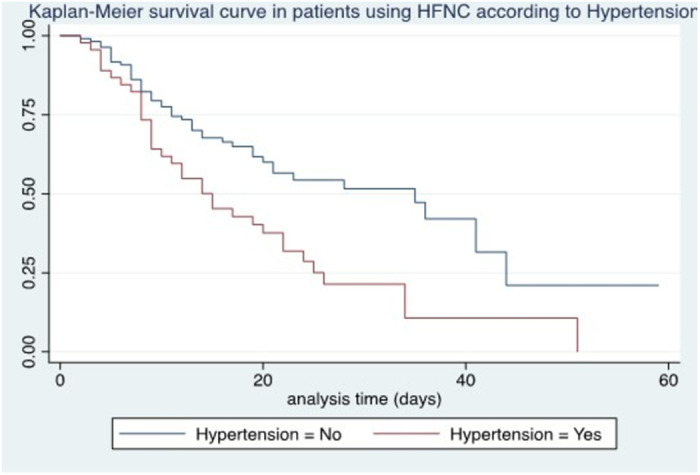
Kaplan-Meier survival curve in patients using HFNC according to hypertension.

Survival at 7 days in those older than 60 years was 75% compared to those younger than 60 years with 94.8%. Age older than 60 years was associated with lower survival (p < 0.0001) (Fig 1). Male sex was not associated with lower survival (p = 0.56). A history of hypertension was associated with mortality (p < 0.0015), with 10-day survival being 61.2% vs. 77.5%, depending on the presence or absence of comorbidity (Fig 2). No association was found with diabetes mellitus.

**Fig 3:**
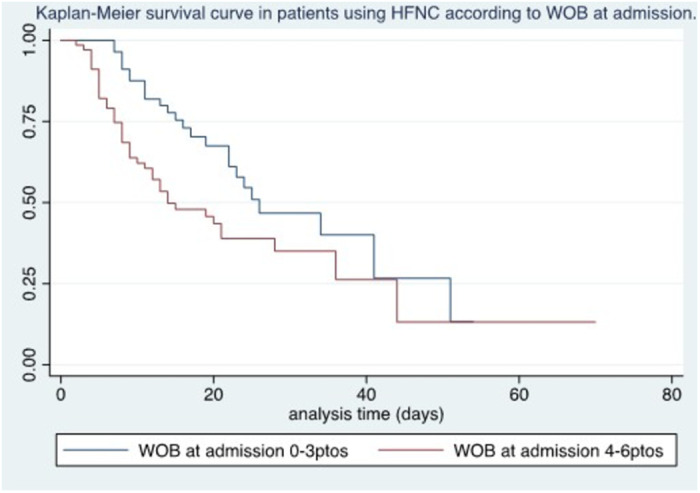
Kaplan-Meier survival curve in patients using HFNC according to WOB at admission.

Among the clinical variables, no association was found between survival with SatO2/FiO2 on admission (p = 0.08) and a WOB scale or respiratory effort ≥ 4 points (p = 0.02) (Fig 3).

**Fig 4:**
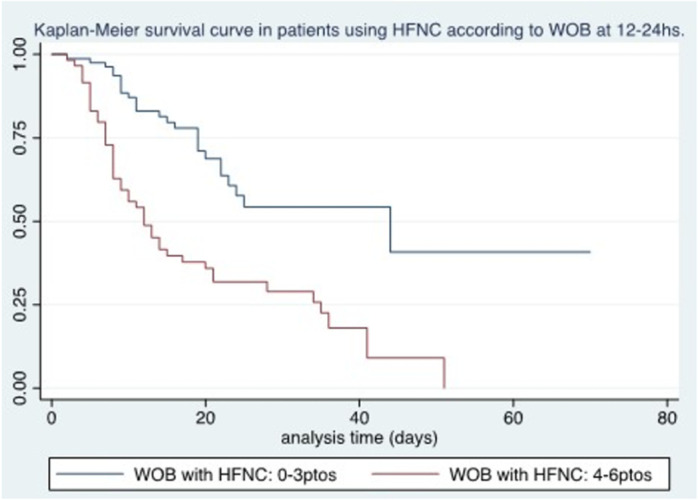
Kaplan-Meier survival curve in patients using HFNC according to WOB at 12-24 hours.

**Fig 5:**
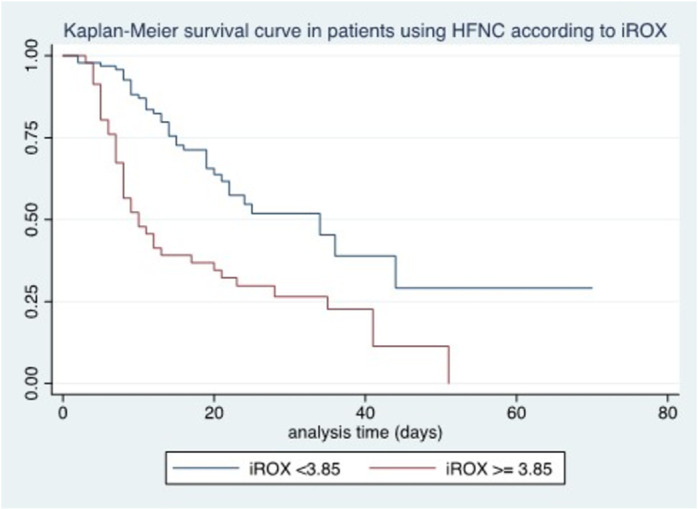
Kaplan-Meier survival curve in patients using HFNC according to iROX.

**Fig 6:**
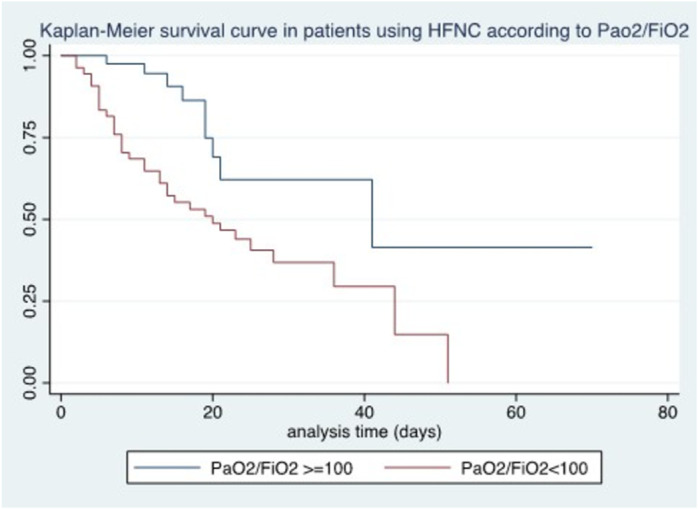
Kaplan-Meier survival curve in patients using HFNC according to PaO2/FiO2.

Among the oxygen and ventilatory variables related to treatment with HFNC 12–24 hours after initiation of treatment, we found an association between survival and SatO2/FiO2 (p < 0.04), WOB ≥4 points (p < 0.0001), iROX < 3.85 (p < 0.0001), and PaO2/FiO2 < 100 (p < 0.002) (Figs 4–6).

**Fig 7:**
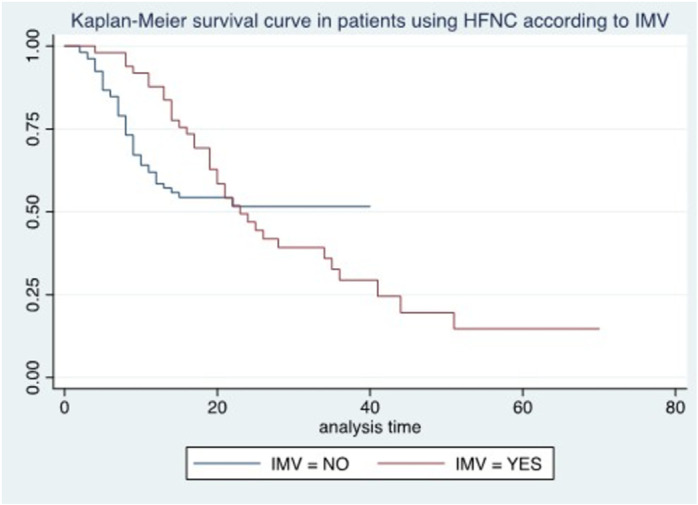
Kaplan-Meier survival curve in patients using HFNC according to IMV.

No patient without mechanical ventilation criteria died; survival in those with mechanical ventilation criteria was 60.9% at 10 days and 45.6% at 15 days. While patients who used mechanical ventilation had a survival rate of 75.6% at 15 days compared to those who did not use mechanical ventilation at 54.3%, in the latter, the overall survival was not less than 51.4% at the end of the study. There was no association with survival (p = 0.46) (Fig 7).

### Simple and multiple Cox analyses

A simple Cox analysis was performed, and an association was found between survival and age ≥ 60 years, arterial hypertension as a comorbidity, WOB ≥ 4 points at hospital admission, lymphocytes < 10%, WOB ≥ 4 points with the use of HFNC, iROX < 3.85, and PaO2/FiO2 < 100. No association was found with SatO2/FiO2 (p = 0.063). An association was found between survival and time on mechanical ventilation but was not reported in the Cox analysis because it did not meet proportionality of hazards; an association was also found with CRP values but was not reported because its categorization did not meet proportionality of hazards (Table 3).

**Table 3:** Cox analysis.

Risk Factor	HR crude	P value	HR adjusted	P value	[95% confidence interval]	
Age ≥ 60 years	2.59	<0.001	2.23	0.010	1.21	4.08
Arterial hypertension	2.01	0.002	1.87	0.045	1.01	3.45
WOB at admission ≥ 4 points	1.83	0.019	2.38	0.004	1.31	4.35
Lymphocytes <10%	1.90	0.015	1.45	0.23	0.79	2.63
iROX < 3.85	2.57	<0.001	1.71	0.047	1.01	2.93
PaO2/FiO2 < 100	2.98	0.004	–	–	–	–
WOB with HFNC ≥ 4 points	2.97	<0.001	–	–	–	–

Multivariate crude and adjusted analysis using the Cox test, HR: hazard ratio, WOB: work of breathing, iROX: ROX index.

Multiple Cox analyses included age, hypertension, WOB ≥ 4 points at hospital admission, and lymphocytes < 10%. Treatment variables or parameters were considered to be entered as iROX. WOB ≥ 4 points with the use of HFNC and PaO2/FiO2 < 100 were not considered as they had sub-variables included in the iROX. The collinearity of variables and proportionality were assessed to detail the final model with prior assessment of independence and survival censorship.

The result shows that the variables that decrease survival in patients with COVID-19 HFNC users are age ≥ 60 years [HR = 2.23 (1.21–4.08)], presence of hypertension [HR = 1.87 (1.01–3.45)], WOB at hospital admission [HR = 2.38 (1.31–4.35)], and iROX < 3.85 [HR = 1.71 (1.01–2.93)], independent of lymphocytes < 10% (Table 3).

## Discussion

Respiratory support methods have become a widely used treatment for the management of hypoxemic respiratory failure during and after the COVID-19 pandemic. And while prior to the onset of the COVID-19 pandemic, the use of HFNC offered benefits in terms of mortality and orotracheal intubation rates [20], ARDS did not present the same criteria, and different pathologies leading to ARDS were addressed [21].

A comparison of the efficacy on mortality between NIV, HFNC, and conventional oxygen therapy may consider HFNC more likely to be the better therapy, according to a systematic review by Pisciotta et al. [21], as well as offering longer ventilator-free days but no improvement in ICU stay [22].

### Overall survival and analysis of IMV use

The present study, conducted in a Peruvian population during the second wave of the COVID-19 pandemic, at a time when immunization was not widespread and only for health care personnel, found that more than 60% of patients had failure in the use of HFNC, mortality occurred in more than half of the patients, and survival in patients using HFNC was less than 50% at 30 days and 16% at the end of the study. Numerous studies describe large variations in outcomes, such as failure of HFNC from 28.8% to 62.5%, the latter in the Italian population, and in mortality from 16% to 80%, the latter in the Chinese population [23]. Many of these variations are explained by the sample size of the studies, the design, timing, and location of the use of these devices, which are related to the waves that occurred during the pandemic and the preparedness of health systems.

Although more than 60% of patients had HFNC failure, more than half of these were not admitted to mechanical ventilation, and only 32% of the entire population received mechanical ventilation; however, mortality in those who used mechanical ventilation was 68%, much higher than that reported in the entire study population. An analysis of the implication of this variable’s time to orotracheal intubation and time on mechanical ventilation on the overall survival of the population could not be performed. However, there does not appear to be any difference between early vs. late time to orotracheal intubation in overall mortality nor in mortality assessed at 14 and 28 days of time to orotracheal intubation or use of HFNC [24].

### Analysis of the prognostic model

It could be determined that in the COVID-19 hospitalized population using HFNC, the hazard of mortality increases by more than 1.5 times in patients with arterial hypertension and iROX < 3.85 and by more than 2 times in patients older than 60 years and in those with a WOB scale at admission ≥ 4 points. This has some similarities with the study by Chandel et al., who, based on a small population size, found that in patients with the use of HFNC and subsequent use of mechanical ventilation, age was associated with mortality; furthermore [24], Alvarez A. et al., in a Spanish population in which ventilatory support was given by HFNC or NIV, found that comorbidities such as diabetes and high creatinine values 3 days after admission to the ICU were associated with mortality, and high PaO2/FiO2 values decreased the risk of mortality [25]. Innocenti et al. also found that scores on the HACOR scale (sub-variables PaO2/FiO2 and respiratory rate) higher than 5 at 2 days after initiation of NIV and age were associated with higher in-hospital mortality [19].

### Analysis of oxygenation parameters

Studies such as that of Chandel et al, consider iROX as a variable that could predict early or late HFNC failure and the need for mechanical ventilation with values >3 at 2, 6 and 12 hours, but it did not predict mortality [24] and other studies have not been able to demonstrate its prediction of mortality at day 0 and 2 after the use of NIV [19], which does not coincide with our results where the role of iROX<3. 85 is important as a predictor of mortality in the survival and multivariate analysis, probably because its measurement was performed between 12 and 24 hours after the start of HFNC when SatO2 values are more stable, and also because NIV was not used as a treatment strategy.

Nevola et al. demonstrated that PaO2/FiO2 values <= 100 predicted in-hospital mortality and days of ICU stay in patients with NIV [26], and PaO2/FiO2 and lactate were associated with 90-day mortality in users of NIV [27]. This partially coincides with our findings in that although these oxygenation variables of HFNC use were measured between 12 and 24 hours after use and the population was larger in our study, the PaO2/FiO2 value could not be determined in the multivariate model due to a significant number of missing data and the preference for using other oxygenation variables such as iROX.

Our study performed measurements of the WOB scale for respiratory effort before and after the initiation of HFNC. Although this scale has been standardized for use and monitoring of treatment with HFNC [28], its variables, such as respiratory frequency and accessory muscle use, have a proportional relationship, and therefore we measured them prior to the use of HFNC with a score ≥4 points, which is related to a greater use of accessory muscles and therefore a possible need for mechanical ventilation [29]. Although there are studies such as Innocenti et al. in which the HACOR scale has respiratory frequency as a variable, with a score >5 at 48 hours after initiation of NIV being associated with mortality [19], the present study is one of the first to evaluate a respiratory effort variable before and after the use of HFNC and also to reflect the importance of the WOB scale on admission and the assessment of respiratory effort in the prediction of in-hospital mortality.

### Clinical implications

The present study helps us to understand the characteristics of the Peruvian population that required HFNC as a treatment strategy during the second wave of COVID-19, where we found a population admitted with ARF to the hospitalization units, and in which we observed that although mortality was higher than half of the population, it was possible to improve survival with the use of HFNC, which served as a basis for its continued use in subsequent waves, for its assessment of its effectiveness in requiring mechanical ventilation or NIV, This served as a basis for continued use in subsequent waves for the evaluation of its effectiveness in requiring mechanical ventilation, NIV, or mortality in other diseases causing ARF or other acute or chronic processes [30]. It also helps us to understand the epidemiological variables involved, such as age [26,27] and chronic comorbidities such as arterial hypertension, diabetes mellitus, and obesity, which are important in the approach to these patients with HFNC or NIV as first-line therapy in the vast majority of studies during the first waves of the pandemic [25], and even multimorbidity scales such as the Charlson index [19]. Additionally, it shows the importance of oxygenation parameters in predicting survival with iROX, even in the assessments on admission prior to the use of HFNC, such as the respiratory effort scale, which aims to give importance to the clinical condition of our patient for a more adequate assessment of the first-line treatment options. The strength of the present study lies in these assessments and their potential use in subsequent prospective studies and clinical trials.

### Limitations

Our study has several limitations. Firstly, it is a retrospective, single-center study in which the population that used HFNC had an average time of illness of 7 days, and most of them arrived in ARF [31]. This being a predictor of HFNC use, this occurred during the second wave, and therefore the results cannot be generalized; however, they provide us with valuable information about the type of population with severe disease that was managed with this device, their outcomes, and the associated factors in a time prior to vaccination. In addition, the sampling considered was non-probabilistic, which could have altered the internal validity of the study due to the lack of randomization. Second, there are several missing data that may have relative importance to the oxygenation parameters in the univariate and multivariate analyses, and although a simple imputation was performed, the distribution of the data may be altered. Third, effective adjuvant treatments such as prone position are not considered [32], and although the vast majority of the older adult population started the immunization process with its real effects in the second and third trimesters of 2021 [33], vaccination status was not considered as a variable; therefore, the information provided helps us assess risk factors in a non-immunized population during the pandemic. Fourth, in the multivariate analysis, invasive mechanical ventilation was not considered an intervening variable due to the proportionality criterion, so the real impact of this variable and associated complications such as pneumothorax, ventilator-associated pneumonia, acute kidney injury, or venous thromboembolism on mortality could not be seen; however, it allows us to take a more valid approach to multivariate analysis considering all the assumptions for its implementation [24].

### Strengths

Our study has multiple strengths. Firstly, this research considers a wide range of clinical and oxygenation parameters both at hospital admission and during treatment with HFNC. This breadth of data allows for a comprehensive assessment of factors associated with survival, which adds robustness to the study’s conclusions. In addition, multivariate analysis identified independent factors associated with survival, strengthening the robustness of the conclusions drawn. The findings on the association of age, the presence of comorbidities such as hypertension, and parameters such as WOB and iROX with survival have high clinical relevance and may influence medical practice and clinical decision-making. In addition, the study was also conducted in the context of the second wave of the COVID-19 pandemic, a critical period with high demand for resources and hospital care. This adds relevance to the results, reflecting the reality of the health crisis and providing valuable information on the use of HFNC during this period. Finally, by drawing on data from actual clinical practice during a global health crisis, the study makes a significant contribution to the existing scientific literature. Furthermore, the results reflect the relevance of HFNC use in a real-world setting, providing a practical and applicable perspective that can guide future research in the management of ARF, not only in the context of COVID-19 but also for other emerging viruses that could cause outbreaks or pandemics. Understanding the efficacy of HFNC and identifying key prognostic factors may be valuable in addressing health emergencies caused by outbreaks and pandemics caused by other pathogens, facilitating a more effective response, and improving outcomes for patients with ARF in similar contexts.

## Conclusions

In-hospital mortality was 51.6% in patients using HFNC who were mostly admitted for respiratory failure to general hospitalization units. Survival at 14 days was 62%, and at the end of follow-up, it was 16.3% in the population studied in the period March to May 2021.

Factors associated with survival were hypertension and iROX < 3.85 with a 1.5-fold mortality hazard, age over 60 years, and respiratory effort scale on admission WOB ≥ 4 points with more than two times the mortality hazard.

These findings highlight the importance of assessing both comorbidities and specific clinical parameters at admission in predicting survival in patients with ARF receiving HFNC. Early identification of these factors may be relevant to personalizing clinical management and improving outcomes in this patient population.
