# Supplementary material for: Factors associated with the survival of adults with COVID-19 using a high-flow nasal cannula in a tertiary hospital in northern Peru during the second wave of the pandemic
Source: PLoS One. 2025 Apr 16;20(4):e0309855. doi: 10.1371/journal.pone.0309855 (PMC12002505; doi:10.1371/journal.pone.0309855)
Supplement: Attachment — Submitted filename: Response to Reviewers.pdf [file pone.0309855.s002.pdf]

Dear **Editor**,

Thank you very much for reviewing our article, " ***Factors associated with the survival of adults with COVID-19 using a high-flow nasal cannula in a tertiary hospital in northern Peru during the second wave of the pandemic***". Your suggestions and comments will be addressed below. Thank you for your valuable time and excellent review.

Editor's comments

Our response: Thank you very much for the comments, they have been reviewed properly.

- The English grammar was reviewed and corrected.
- The modifications suggested by the reviewers were implemented.
- Authors' affiliations and scientific names were verified.
- The platform was updated according to the changes made.

Reviewer #1:

The article addresses topics related to factors associated with the survival of adults with COVID-19 using a high-flow nasal cannula in a tertiary hospital during the second wave of the pandemic in Peru. They have an interesting approach. In this regard, I have some recommendations:
Our response: Thanks for your comments

1. **Reviewer says:** Introduction: I suggest that, in terms of novelty and relevance, the justification for the study should be better supported.
   Our response: An additional paragraph was added in the introduction about the scarcity of Peruvian studies, and the importance of the study was modified.

2. **Reviewer says:** Methodology: The authors should detail whether the data meet the statistical assumptions to be able to execute the Cox regression. Additionally, given the variables included in the multivariate analysis, it would be ideal to evaluate multicollinearity.
   Our response: The assumptions are specified in the methodology and results.

3. **Reviewer says:** Discussion: Delve deeper into the potential impact of the limitations and, especially, what were the strategies to mitigate said impact.
   Our response: "The limitations are specified and the impact of these is determined, as well as the way to address some of them.

Reviewer #2:

Title: Factors associated with the survival of adults with COVID-19 using a high-flow nasal cannula in a tertiary hospital in northern Peru during the second wave of the pandemic. Is more appropriate to change survival to mortality in the title:
Our response: "Although the studies do not consider whether it is better to evaluate mortality or survival in these types of designs, our manuscript aims to evaluate survival, and although the initial descriptive tables consider the term mortality, the curves being evaluated are survival curves and not mortality risk, and the Cox analysis allows us to analyze our general objective and find results based on that outcome".

1. **Reviewer says:** 1- Abstract: During March and May to change to during March to May and other sentences use word during March and May
   Our response: "The change has been made in the manuscript".

2. **Reviewer says:** 2- Methodology: Authors need to add hospital bed admission data especially ICU admission. How many data been excluded during the study period and reason for excluding.
   Our response: It is described that the patients evaluated were those who were admitted to a general ward and/or ICU of the hospital. It is worth noting that only those who required mechanical ventilation were transferred to the ICU, while the rest were managed in the general ward under the supervision of critical care. This is detailed in procedures and techniques.

3. **Reviewer says:** 3- Results: Need to follow the tables and figure format of journal.
   Our response: Thank you for your comment. The editor has given the green light.

4. **Reviewer says:** 4- Analysis: age >=60 or age > 60 is the correct group. 57.8 ± 14.8 lt/min. or L/min, WOB >=4 to replace with another symbol
   Our response: This was modified in the manuscript

5. **Reviewer says:** Discussion: variable—time, ventilation—on why dash is long.
   Our response: This was modified in the manuscript

If you have any comments or recommendations, we are ready to respond.

Sincerely,

Mario J. Valladares-Garrido

Universidad Continental, Lima 15046, Peru; mvalladares@continental.edu.pe
