# Supplementary material for: Factors associated with the survival of adults with COVID-19 using a high-flow nasal cannula in a tertiary hospital in northern Peru during the second wave of the pandemic
Source: PLoS One. 2025 Apr 16;20(4):e0309855. doi: 10.1371/journal.pone.0309855 (PMC12002505; doi:10.1371/journal.pone.0309855)
Supplement: Attachment — Submitted filename: Response to Editor.docx [file pone.0309855.s001.docx]

Dear editor, it was modified according to your recommendations.

(1) Before we can proceed, please report in the Methods section the dates when data were accessed for research purposes(dd/mm/yyyy). Please note that if this information is not included when your manuscript is resubmitted, it may be rejected.

**Our response:**

We have added the recommendations on the dates on which the data was accessed in the process and methodology techniques section.

Data collection started on August 22, 2023, and continued until February 10, 2024, using the data collection form with the previously developed database.

We have resolved the observations. If you have any questions, we will be delighted to answer them.

Sincerely,

**Mario J. Valladares-Garrido**

Universidad Continental, Lima 15046, Peru; [mvalladares@continental.edu.pe](mailto:mvalladares@continental.edu.pe)
