# Supplementary material for: Factors associated with the survival of adults with COVID-19 using a high-flow nasal cannula in a tertiary hospital in northern Peru during the second wave of the pandemic
Source: PLoS One. 2025 Apr 16;20(4):e0309855. doi: 10.1371/journal.pone.0309855 (PMC12002505; doi:10.1371/journal.pone.0309855)
Supplement: Peer review history

# Author comment: Author response to Decision Letter 0

**DOI:** 10.1371/journal.pone.0309855.r001

22 Aug 2024

Attachment Submitted filename: Response to Editor.docx

# Peer review: Decision Letter 0

**DOI:** 10.1371/journal.pone.0309855.r002

**Authors:** Tai-Heng Chen

8 Dec 2024

PONE-D-24-35518Factors associated with the survival of adults with COVID-19 using a high-flow nasal cannula in a tertiary hospital in northern Peru during the second wave of the pandemic.PLOS ONE

Dear Dr. Valladares-Garrido,

Thank you for submitting your manuscript to PLOS ONE. After careful consideration, we feel that it has merit but does not fully meet PLOS ONE’s publication criteria as it currently stands. Therefore, we invite you to submit a revised version of the manuscript that addresses the points raised during the review process.

Please submit your revised manuscript by Jan 22 2025 11:59PM. If you will need more time than this to complete your revisions, please reply to this message or contact the journal office at plosone@plos.org . When you're ready to submit your revision, log on to https://www.editorialmanager.com/pone/ and select the 'Submissions Needing Revision' folder to locate your manuscript file.

Please include the following items when submitting your revised manuscript:

- A rebuttal letter that responds to each point raised by the academic editor and reviewer(s). You should upload this letter as a separate file labeled 'Response to Reviewers'.
- A marked-up copy of your manuscript that highlights changes made to the original version. You should upload this as a separate file labeled 'Revised Manuscript with Track Changes'.
- An unmarked version of your revised paper without tracked changes. You should upload this as a separate file labeled 'Manuscript'.

If you would like to make changes to your financial disclosure, please include your updated statement in your cover letter. Guidelines for resubmitting your figure files are available below the reviewer comments at the end of this letter.

If applicable, we recommend that you deposit your laboratory protocols in protocols.io to enhance the reproducibility of your results. Protocols.io assigns your protocol its own identifier (DOI) so that it can be cited independently in the future. For instructions see: https://journals.plos.org/plosone/s/submission-guidelines#loc-laboratory-protocols . Additionally, PLOS ONE offers an option for publishing peer-reviewed Lab Protocol articles, which describe protocols hosted on protocols.io. Read more information on sharing protocols at https://plos.org/protocols?utm_medium=editorial-email&utm_source=authorletters&utm_campaign=protocols .

We look forward to receiving your revised manuscript.

Kind regards,

Tai-Heng Chen, M.D., Ph.D.

Academic Editor

PLOS ONE

Journal requirements:

When submitting your revision, we need you to address these additional requirements.

1. Please ensure that your manuscript meets PLOS ONE's style requirements, including those for file naming. The PLOS ONE style templates can be found at 

https://journals.plos.org/plosone/s/file?id=wjVg/PLOSOne_formatting_sample_main_body.pdf and 

https://journals.plos.org/plosone/s/file?id=ba62/PLOSOne_formatting_sample_title_authors_affiliations.pdf

2. Please include captions for your Supporting Information files at the end of your manuscript, and update any in-text citations to match accordingly. Please see our Supporting Information guidelines for more information: http://journals.plos.org/plosone/s/supporting-information. 

Reviewers' comments:

Reviewer's Responses to Questions

**Comments to the Author**

1. Is the manuscript technically sound, and do the data support the conclusions?

The manuscript must describe a technically sound piece of scientific research with data that supports the conclusions. Experiments must have been conducted rigorously, with appropriate controls, replication, and sample sizes. The conclusions must be drawn appropriately based on the data presented. 

Reviewer #1: Yes

Reviewer #2: Yes

**********

2. Has the statistical analysis been performed appropriately and rigorously? 

Reviewer #1: Yes

Reviewer #2: Yes

**********

3. Have the authors made all data underlying the findings in their manuscript fully available?

The PLOS Data policy requires authors to make all data underlying the findings described in their manuscript fully available without restriction, with rare exception (please refer to the Data Availability Statement in the manuscript PDF file). The data should be provided as part of the manuscript or its supporting information, or deposited to a public repository. For example, in addition to summary statistics, the data points behind means, medians and variance measures should be available. If there are restrictions on publicly sharing data—e.g. participant privacy or use of data from a third party—those must be specified.

Reviewer #1: Yes

Reviewer #2: Yes

**********

4. Is the manuscript presented in an intelligible fashion and written in standard English?

PLOS ONE does not copyedit accepted manuscripts, so the language in submitted articles must be clear, correct, and unambiguous. Any typographical or grammatical errors should be corrected at revision, so please note any specific errors here.

Reviewer #1: Yes

Reviewer #2: Yes

**********

5. Review Comments to the Author

Please use the space provided to explain your answers to the questions above. You may also include additional comments for the author, including concerns about dual publication, research ethics, or publication ethics. (Please upload your review as an attachment if it exceeds 20,000 characters)

Reviewer #1: The article addresses topics related to factors associated with the survival of adults with COVID-19 using a high-flow nasal cannula in a tertiary hospital during the second wave of the pandemic in Peru. They have an interesting approach. In this regard, I have some recommendations:

Introduction: I suggest that, in terms of novelty and relevance, the justification for the study should be better supported.

Methodology: The authors should detail whether the data meet the statistical assumptions to be able to execute the Cox regression. Additionally, given the variables included in the multivariate analysis, it would be ideal to evaluate multicollinearity.

Discussion: Delve deeper into the potential impact of the limitations and, especially, what were the strategies to mitigate said impact.

Reviewer #2: Title: Factors associated with the survival of adults with COVID-19 using a high-flow nasal

cannula in a tertiary hospital in northern Peru during the second wave of the pandemic. Is is more appropriate to change survival to mortality in the title.

1- Abstract: During March and May to change to during March to May and other sentences use word during March and May

2- Methodology: Authors need to add hospital bed admission data especially ICU admission. How many data been excluded during the study period and reason for excluding.

3- Results: Need to follow the tables and figure format of journal.

4- Analysis: age >=60 or age > 60 is the correct group. 57.8 ± 14.8 lt/min. or L/min, WOB >=4 to replace with another symbol

Discussion: variable—time, ventilation—on why dash is long.

**********

6. PLOS authors have the option to publish the peer review history of their article (what does this mean? ). If published, this will include your full peer review and any attached files.

If you choose “no”, your identity will remain anonymous but your review may still be made public.

**Do you want your identity to be public for this peer review?** For information about this choice, including consent withdrawal, please see our Privacy Policy .

Reviewer #1: **Yes: ** Fernando M. Runzer-Colmenares

Reviewer #2: No

**********

[NOTE: If reviewer comments were submitted as an attachment file, they will be attached to this email and accessible via the submission site. Please log into your account, locate the manuscript record, and check for the action link "View Attachments". If this link does not appear, there are no attachment files.]

While revising your submission, please upload your figure files to the Preflight Analysis and Conversion Engine (PACE) digital diagnostic tool, https://pacev2.apexcovantage.com/ . PACE helps ensure that figures meet PLOS requirements. To use PACE, you must first register as a user. Registration is free. Then, login and navigate to the UPLOAD tab, where you will find detailed instructions on how to use the tool. If you encounter any issues or have any questions when using PACE, please email PLOS at figures@plos.org . Please note that Supporting Information files do not need this step.

# Author comment: Author response to Decision Letter 1

**DOI:** 10.1371/journal.pone.0309855.r003

10 Dec 2024

Dear Editor,

Thank you very much for reviewing our article, " Factors associated with the survival of adults with COVID-19 using a high-flow nasal cannula in a tertiary hospital in northern Peru during the second wave of the pandemic". Your suggestions and comments will be addressed below. Thank you for your valuable time and excellent review.

Editor's comments

Our response: Thank you very much for the comments, they have been reviewed properly.

• The English grammar was reviewed and corrected.

• The modifications suggested by the reviewers were implemented.

• Authors' affiliations and scientific names were verified.

• The platform was updated according to the changes made.

Reviewer #1:

The article addresses topics related to factors associated with the survival of adults with COVID-19 using a high-flow nasal cannula in a tertiary hospital during the second wave of the pandemic in Peru. They have an interesting approach. In this regard, I have some recommendations:

Our response: Thanks for your comments

1. Reviewer says: Introduction: I suggest that, in terms of novelty and relevance, the justification for the study should be better supported.

Our response: An additional paragraph was added in the introduction about the scarcity of Peruvian studies, and the importance of the study was modified.

2. Reviewer says: Methodology: The authors should detail whether the data meet the statistical assumptions to be able to execute the Cox regression. Additionally, given the variables included in the multivariate analysis, it would be ideal to evaluate multicollinearity.

Our response: The assumptions are specified in the methodology and results.

3. Reviewer says: Discussion: Delve deeper into the potential impact of the limitations and, especially, what were the strategies to mitigate said impact.

Our response: “The limitations are specified and the impact of these is determined, as well as the way to address some of them.

Reviewer #2:

Title: Factors associated with the survival of adults with COVID-19 using a high-flow nasal cannula in a tertiary hospital in northern Peru during the second wave of the pandemic. Is more appropriate to change survival to mortality in the title:

Our response: “Although the studies do not consider whether it is better to evaluate mortality or survival in these types of designs, our manuscript aims to evaluate survival, and although the initial descriptive tables consider the term mortality, the curves being evaluated are survival curves and not mortality risk, and the Cox analysis allows us to analyze our general objective and find results based on that outcome”.

1. Reviewer says: 1- Abstract: During March and May to change to during March to May and other sentences use word during March and May

Our response: “The change has been made in the manuscript”.

2. Reviewer says: 2- Methodology: Authors need to add hospital bed admission data especially ICU admission. How many data been excluded during the study period and reason for excluding.

Our response: It is described that the patients evaluated were those who were admitted to a general ward and/or ICU of the hospital. It is worth noting that only those who required mechanical ventilation were transferred to the ICU, while the rest were managed in the general ward under the supervision of critical care. This is detailed in procedures and techniques.

3. Reviewer says: 3- Results: Need to follow the tables and figure format of journal.

Our response: Thank you for your comment. The editor has given the green light.

4. Reviewer says: 4- Analysis: age >=60 or age > 60 is the correct group. 57.8 ± 14.8 lt/min. or L/min, WOB >=4 to replace with another symbol

Our response: This was modified in the manuscript

5. Reviewer says: Discussion: variable—time, ventilation—on why dash is long.

Our response: This was modified in the manuscript

If you have any comments or recommendations, we are ready to respond.

Sincerely,

Mario J. Valladares-Garrido

Universidad Continental, Lima 15046, Peru; mvalladares@continental.edu.pe

Attachment Submitted filename: Response to Reviewers.pdf

# Peer review: Decision Letter 1

**DOI:** 10.1371/journal.pone.0309855.r004

**Authors:** Tai-Heng Chen

5 Feb 2025

Factors associated with the survival of adults with COVID-19 using a high-flow nasal cannula in a tertiary hospital in northern Peru during the second wave of the pandemic.

PONE-D-24-35518R1

Dear Dr. Valladares-Garrido,

We’re pleased to inform you that your manuscript has been judged scientifically suitable for publication and will be formally accepted for publication once it meets all outstanding technical requirements.

Within one week, you’ll receive an e-mail detailing the required amendments. When these have been addressed, you’ll receive a formal acceptance letter and your manuscript will be scheduled for publication.

An invoice will be generated when your article is formally accepted. Please note, if your institution has a publishing partnership with PLOS and your article meets the relevant criteria, all or part of your publication costs will be covered. Please make sure your user information is up-to-date by logging into Editorial Manager at Editorial Manager®  and clicking the ‘Update My Information' link at the top of the page. If you have any questions relating to publication charges, please contact our Author Billing department directly at authorbilling@plos.org.

If your institution or institutions have a press office, please notify them about your upcoming paper to help maximize its impact. If they’ll be preparing press materials, please inform our press team as soon as possible -- no later than 48 hours after receiving the formal acceptance. Your manuscript will remain under strict press embargo until 2 pm Eastern Time on the date of publication. For more information, please contact onepress@plos.org.

Kind regards,

Tai-Heng Chen, M.D., Ph.D.

Academic Editor

PLOS ONE

Reviewers' comments:

Reviewer's Responses to Questions

**Comments to the Author**

1. If the authors have adequately addressed your comments raised in a previous round of review and you feel that this manuscript is now acceptable for publication, you may indicate that here to bypass the “Comments to the Author” section, enter your conflict of interest statement in the “Confidential to Editor” section, and submit your "Accept" recommendation.

Reviewer #1: All comments have been addressed

Reviewer #2: All comments have been addressed

**********

2. Is the manuscript technically sound, and do the data support the conclusions?

The manuscript must describe a technically sound piece of scientific research with data that supports the conclusions. Experiments must have been conducted rigorously, with appropriate controls, replication, and sample sizes. The conclusions must be drawn appropriately based on the data presented. 

Reviewer #1: Yes

Reviewer #2: Yes

**********

3. Has the statistical analysis been performed appropriately and rigorously? 

Reviewer #1: Yes

Reviewer #2: Yes

**********

4. Have the authors made all data underlying the findings in their manuscript fully available?

The PLOS Data policy requires authors to make all data underlying the findings described in their manuscript fully available without restriction, with rare exception (please refer to the Data Availability Statement in the manuscript PDF file). The data should be provided as part of the manuscript or its supporting information, or deposited to a public repository. For example, in addition to summary statistics, the data points behind means, medians and variance measures should be available. If there are restrictions on publicly sharing data—e.g. participant privacy or use of data from a third party—those must be specified.

Reviewer #1: Yes

Reviewer #2: Yes

**********

5. Is the manuscript presented in an intelligible fashion and written in standard English?

PLOS ONE does not copyedit accepted manuscripts, so the language in submitted articles must be clear, correct, and unambiguous. Any typographical or grammatical errors should be corrected at revision, so please note any specific errors here.

Reviewer #1: Yes

Reviewer #2: Yes

**********

6. Review Comments to the Author

Please use the space provided to explain your answers to the questions above. You may also include additional comments for the author, including concerns about dual publication, research ethics, or publication ethics. (Please upload your review as an attachment if it exceeds 20,000 characters)

Reviewer #1: No more comments. The authors addressed the observations correctly and the article improved substantially. It is ready to be published.

Reviewer #2: Congratulations

Suggestions: It would be better if authors can describe the number of patients screened for study based on total number of ICU admissions during the study period. And explain how many patients been excluded in the flow chart.

**********

7. PLOS authors have the option to publish the peer review history of their article (what does this mean? ). If published, this will include your full peer review and any attached files.

If you choose “no”, your identity will remain anonymous but your review may still be made public.

**Do you want your identity to be public for this peer review?** For information about this choice, including consent withdrawal, please see our Privacy Policy .

Reviewer #1: **Yes: ** Fernando M. Runzer-Colmenares

Reviewer #2: No

**********

# Editor report: Acceptance letter

**DOI:** 10.1371/journal.pone.0309855.r005

**Authors:** Tai-Heng Chen

PONE-D-24-35518R1

PLOS ONE

Dear Dr. Valladares-Garrido,

I'm pleased to inform you that your manuscript has been deemed suitable for publication in PLOS ONE. Congratulations! Your manuscript is now being handed over to our production team.

At this stage, our production department will prepare your paper for publication. This includes ensuring the following:

* All references, tables, and figures are properly cited

* All relevant supporting information is included in the manuscript submission,

* There are no issues that prevent the paper from being properly typeset

If revisions are needed, the production department will contact you directly to resolve them. If no revisions are needed, you will receive an email when the publication date has been set. At this time, we do not offer pre-publication proofs to authors during production of the accepted work. Please keep in mind that we are working through a large volume of accepted articles, so please give us a few weeks to review your paper and let you know the next and final steps.

Lastly, if your institution or institutions have a press office, please let them know about your upcoming paper now to help maximize its impact. If they'll be preparing press materials, please inform our press team within the next 48 hours. Your manuscript will remain under strict press embargo until 2 pm Eastern Time on the date of publication. For more information, please contact onepress@plos.org.

If we can help with anything else, please email us at customercare@plos.org.

Thank you for submitting your work to PLOS ONE and supporting open access.

Kind regards,

PLOS ONE Editorial Office Staff

on behalf of

Dr. Tai-Heng Chen

Academic Editor

PLOS ONE
